# Hashimoto’s thyroiditis in an Egyptian cohort: clinical, functional, and ultrasonographic features with insights into nodule risk

**DOI:** 10.3389/fendo.2025.1664047

**Published:** 2025-11-06

**Authors:** Aliaa El Aghoury, Samar Abd ElHafeez, Basma El Sabaa, Reham Abo El Wafa, Waleed Abo El Wafa, Hanaa El Naggar, Eiman Ibrahim, Rania Naguib, Maha Bondok

**Affiliations:** 1 Department of Endocrinology and Metabolism, Faculty of Medicine, Alexandria University, Alexandria, Egypt; 2 Department of Epidemiology, High Institute of Public Health, Alexandria University, Alexandria, Egypt; 3 Department of Pathology, Faculty of Medicine, Alexandria University, Alexandria, Egypt; 4 Department of Clinical and Chemical Pathology, Faculty of Medicine, Alexandria University, Alexandria, Egypt; 5 Department of General Surgery, Endocrine Surgery Unit, Faculty of Medicine, Alexandria University, Alexandria, Egypt; 6 Department of Endocrinology and Metabolism, University of Missouri, Columbia, MO, United States; 7 Department of Internal Medicine, College of Medicine, Princess Nourah bint Abdulrahman University, Riyadh, Saudi Arabia

**Keywords:** Hashimoto thyroiditis (HT), thyroid nodules, nodular Hashimoto thyroiditis, thyroid malignancy, thyroid ultrasonography (US), fine needle aspiration cytology (FNAC)

## Abstract

**Background/aim:**

Hashimoto’s thyroiditis (HT) is a highly prevalent autoimmune disorder. Its coexistence with benign and malignant thyroid nodules is well-documented; however, data from non-Western countries remain limited. Our objectives were to determine the demographics, clinical presentation, biochemical parameters, and thyroid ultrasonographic findings in an Egyptian cohort with HT; estimate nodule prevalence; and identify potential risk factors for nodular presentation.

**Patients and methods:**

A cross-sectional study was conducted on 408 newly diagnosed patients with HT at Alexandria University Hospital. Sociodemographic, clinical presentation, biochemical (thyroid function and autoantibodies thyroperoxidase and thyroglobulin Abs), and ultrasonographic data were collected. Thyroid nodules were classified according to the American College of Radiology Thyroid Imaging Reporting and Data System (ACR TI-RADS). Fine-needle aspiration cytology (FNAC) was classified by the Bethesda system (BSRTC). Multiple logistic regression identified predictors of nodularity.

**Results:**

Among our cohort of 408 participants (female-to-male ratio of 15:1; mean age 38.6 years), 23.5% had thyroid nodules on ultrasound. Nodules were more frequent in those ≥35 years and with a family history of thyroid disease. Compressive symptoms were more common in the nodular group (33.0% vs. 18.6%). Hypothyroidism was observed in 80.9%, predominantly subclinical, and was more frequent in the non-nodular group (80.4% vs. 71.1%). Autoantibodies tested positive in 87.5%. One-third had diffuse enlargement; most nodules were classified as TIRADS 3 or 4. FNAC (*n* = 49) showed 63.2% benign, 32.7% indeterminate, and 4.1% non-diagnostic. Histopathology (*n* = 18) identified papillary thyroid cancer in 44.4%. In multiple logistic regression, age 35–50 (OR = 7.023, 95% CI: 1.447–334.090), age ≥50 (OR = 8.589, 95% CI: 1.740–42.402), family history of goiter/thyroid cancer (OR = 5.177, 95% CI: 1.055–25.403), lower TSH (OR = 0.981, 95% CI: 0.966–0.997), TPOAb (OR = 0.998, 95% CI: 0.997–0.999), and larger thyroid volume (OR = 1.036, 95% CI: 1.012–1.060) were independent predictors of nodularity.

**Conclusion:**

HT shows heterogeneous clinical presentations, with subclinical hypothyroidism predominating. Compressive symptoms are more common in patients with nodules. Ultrasound and FNAC are essential for the management of nodules with HT and can help prevent unnecessary surgery. Older age, larger thyroid volume, and a positive familial history of goiter and/or thyroid cancer are major predictors for nodularity. The malignancy rate is ~2%, with microcalcifications strongly associated with malignancy.

## Introduction

Hashimoto’s thyroiditis (HT), also referred to as chronic lymphocytic or autoimmune thyroiditis, is a highly prevalent endocrine disorder. It predominantly affects women, who are four to eight times more likely than men to develop this condition. The prevalence of HT varies widely across different geographic regions and socioeconomic levels (1, 2).

HT is an organ-specific autoimmune disorder characterized by loss of self-tolerance to thyroid antigens. Serum from patients with HT exhibits elevated levels of autoantibodies, specifically anti-thyroglobulin antibody (TgAb) and thyroperoxidase antibody (TPOAb), and less commonly thyrotropin receptor antibodies (TRAb) (2, 3). Pathologically, HT is known as “struma lymphomatosa” due to its characteristic lesions, which include a large amount of parenchymal infiltration by lymphocytes and plasma cells. This is accompanied by thyroid follicular cell injury, followed by subsequent fibrosis and parenchymal atrophy. These histological features indicate that the thyroid gland was targeted by a combination of cell- and antibody-mediated autoimmune responses (4).

The precise pathogenesis of HT is not fully understood. It is recognized as a complex and progressively expanding disorder, driven by the interaction of genetic susceptibility and environmental factors, all within the context of immune dysregulation. Among these factors, genetic factors are thought to play a significant role in the development of HT (2–4). Recently, novel pathogenic mechanisms have been elucidated, where recent studies support the importance of epigenetics in the initiation and perpetuation of thyroid autoimmunity, particularly under certain environmental factors (5).

HT is predominantly associated with and is the leading cause of hypothyroidism in iodine-sufficient areas. It may initially present with a transient phase of thyrotoxicosis, resulting from the destruction of thyroid follicular cells and the subsequent release of thyroid hormones. The hyperthyroid phase eventually progresses to permanent hypothyroidism. The spectrum of clinical manifestations associated with hypothyroidism is broad, ranging from subtle manifestations to severe life-threatening complications (4).

The diagnosis of hypothyroidism is based on a combination of clinical manifestations and the presence of positive thyroid autoantibodies. However, it is worth noting that seronegative HT accounts for 5%–10% of cases (2, 6). In such cases, ultrasound (US) plays a crucial role in diagnosis. Characteristic US features include the presence of diffuse hypoechoic, heterogeneous echotexture of thyroid parenchyma, often accompanied by a pseudo-nodular appearance and hypervascularity. However, focal thyroiditis, commonly known as nodular HT, can be observed either on a background of diffuse thyroiditis or on a background of normal thyroid parenchyma (7). Nevertheless, the most precise diagnosis of HT continues to be the characteristic histological findings derived from biopsy.

It has been well-established that HT has been associated with both benign and malignant thyroid nodules (8). The presence of HT substantially increases the risk of both papillary thyroid cancer (PTC) and thyroid lymphoma. Notably, the relationship between HT and PTC was first observed by Dailey et al. in 1955 (9). Since then, the link between the two entities has been an extensive area of discussion and controversy (10).

Given the diversity in prevalence, presentation, thyroid status, and ultrasound features among patients with HT, this study aimed to assess the demographics, clinical presentation, biochemical investigations (including thyroid profile and thyroid autoantibodies), and ultrasonographic features of the thyroid among patients with HT attending the Alexandria Main University Hospital. Furthermore, we sought to determine the prevalence of nodules among the studied population and to assess their cytopathological features. Finally, the study aimed to identify potential risk factors associated with the development of nodules.

## Patients and methods

### Study design and settings

A cross-sectional study was conducted at Alexandria Main University Hospital, a tertiary care facility, to recruit a diverse cohort of patients from four governorates (Alexandria, Kafr Elsheikh, Beheira, and Matrouh).

#### Study population

A total of 408 patients, aged 18 years and older, were enrolled in this study. These individuals presented to the endocrinology clinic between 1 July 2020 and 31 January 2021, with manifestations suggestive of thyroid dysfunction or neck swelling, with or without compressive symptoms.

#### Inclusion criteria

Only newly diagnosed patients with HT were included in this study.

Diagnosis of seropositive HT was based on the following criteria:

1. Biochemical affirmation of positive results of the TPOAb and/or TgAb.
2. Ultrasonographic finding suggestive of HT (diffuse hypoechogenicity of the thyroid gland).

Diagnosis of seronegative HT was based on the following criteria (6, 11):

1. The presence of subclinical hypothyroidism was based on a serum TSH value of ≥4.5 mIU/L with normal free thyroxine (FT4), or overt hypothyroidism based on a serum TSH value of ≥4.5 mIU/L with low FT4 levels confirmed on two separate occasions, at least 2 months apart.
2. US finding suggestive of HT (diffuse hypoechogenicity of the thyroid gland).
3. Exclusion of non-autoimmune causes of hypothyroidism.
4. To minimize confounding factors, individuals with a BMI ≥30 kg/m^2^ were excluded only from the seronegative subgroup.

#### Exclusion criteria

Individuals with a history of previous thyroid surgery, radioactive iodine ablation, Graves’ disease, subacute thyroiditis, or use of drugs known to affect thyroid function were excluded from the study. Pregnant and lactating women were also ruled out.

#### Data collection

Sociodemographic factors, including age, sex, marital status, and the number of children, were collected. Additionally, data on family history of autoimmune thyroid disease (manifesting as hypo- or hyperthyroidism), and/or goiter or thyroid cancer, were gathered. The clinical presentation of the enrolled individuals was classified as either asymptomatic or symptomatic. Symptomatic patients were further categorized into those with compressive symptoms related to neck swelling or symptoms indicating thyroid dysfunction, either hypothyroidism (e.g., weight gain, easy fatigability, somnolence, lethargy, cold intolerance, hair fall, depression, constipation, menstrual irregularities in female respondents) or hyperthyroidism (e.g., weight loss, insomnia, tremors, heat intolerance). A detailed clinical examination of the thyroid gland was performed to assess its size, surface, and presence of any palpable nodules or lymph nodes.

Laboratory investigations included the following: 1) thyroid function tests: serum levels of thyroid-stimulating hormone (TSH), free triiodothyronine (FT3), and free thyroxine (FT4) were measured by the fully automated immunoassay analyzer Centaur XP (Siemens, USA) (normal range of TSH: 0.306–4.527 mIU/L, free T3: 2.05–4.36 pg/mL, and free T4: 0.926–1.68 ng/dL; 2) complete lipid profile: total cholesterol, high-density lipoproteins (HDL), low-density lipoproteins (LDL), and triglycerides were measured using a fully automated chemistry analyzer Advia1800 (Siemens, USA; and 3) thyroid autoantibodies: TgAb and TPOAb were measured by the enzyme-linked immunosorbent assay (ELISA). Serum thyrotropin receptor antibodies were tested only in cases with either subclinical or overt hyperthyroidism, to exclude Graves’ disease. The normal range for TgAb was up to 115 IU/mL; for TPOAb, up to 34 IU/mL; and for TRAb, up to 1.75 IU/L.

#### High-resolution ultrasound

Evaluation of the thyroid gland was performed by a single expert sonographer using a commercially available real-time machine (Kontron-imagic Agile) with a 7.5-MHz linear transducer in both transverse and longitudinal planes. A full comment was reported on 1) volume of the thyroid gland using the prolate ellipsoid method (volume = length × breadth × depth × π/6) (thyroid enlargement defined as a volume of ≥18 mL in women and ≥21 mL in men); 2) echogenicity of the thyroid parenchyma as compared to surrounding strap muscles, graded into three grades (grade 1: diffuse hypoechogenicity, still higher than the strap muscles; grade 2: diffuse hypoechogenicity equals to the echogenicity of the strap muscles; and grade 3: diffuse hypoechogenicity less than the echogenicity of the strap muscles; 3) echotexture of the thyroid gland; 4) vascularity of the thyroid parenchyma assessed by color Doppler (classified into no increase, mild, moderate, or marked increase); and 5) the presence of nodules, with detailed comment on size, shape, echogenicity, border, margin, vascularity, calcification, and cervical lymph nodes. The detected nodules were classified based on their sonographic pattern and risk of malignancy, according to the American College of Radiology-Thyroid Imaging Reporting and Data System (ACR TIRADS). Ultrasound-guided fine-needle aspiration cytology was used only in selected cases with thyroid nodules according to the indications of the ACR TIRADS (12). Aspirates from FNA were further assessed by a single expert cytopathologist and categorized according to the 2017 Bethesda pathological classification of thyroid nodules (BSRTC) (13).

- Patients in the study cohort were divided into two groups: those with nodules and those without. A comparison was then made across all examined parameters.
- Patients with thyroid nodules were referred for surgery if they 1) showed obstructive symptoms, 2) had indeterminate cytology based on BSRTC, and 3) some patients opted for surgery in accordance with their preference when offered the choice of follow-up.

### Statistical analysis

Quantitative data were summarized using means ± standard deviations (SDs) or medians with interquartile ranges (IQRs), depending on the distribution of the data. Qualitative data were presented as percentages and frequencies. Comparison of quantitative data was done using an independent *t*-test or a Mann–Whitney *U* test, where appropriate. Chi-square test or Fisher’s exact test was used to test the association between categorical variables.

Multiple logistic regression was employed to identify predictors of the development of thyroid nodules. The model included all potential confounders [age, sex, family history, clinical presentation, TSH, TPOAb, TgAb, and thyroid glandular volume (TGV)] as independent variables and nodular status as the dependent variable. Odds ratios (ORs) with 95% confidence intervals (CIs) were calculated to quantify the strength of the associations. The model’s goodness-of-fit was assessed using the Akaike information criterion (AIC), *R*², and the likelihood ratio test. Then, the data were analyzed using R 4.4.3 software—a nomogram for the parameters associated with thyroid nodules. The nomogram visually represents the ORs with 95% CIs for various predictors of thyroid nodules. The *x*-axis is on a log scale, which facilitates better visualization of variables with large effect sizes. The blue dots represent the OR estimates, while the red error bars indicate the 95% confidence intervals. ORs were derived from multivariable logistic regression, with the *x*-axis presented on a logarithmic scale. An OR greater than 1 indicates increased relative risk of nodularity, whereas an OR less than 1 reflects a protective effect.

#### Ethical considerations

The Ethics Committee of the Faculty of Medicine, Alexandria University, Egypt (IRB No. 0105411), approved the study, following the International Ethical Guidelines for Epidemiological Studies. All participants were given written informed consent after being informed of the nature and aim of the study.

## Results

### Baseline characteristics of the study population

**Table 1** presents the demographic and clinical characteristics of 408 participants, stratified by the presence of thyroid nodules. Specifically, 96 (23.5%) participants had nodules, while 312 (76.5%) had no nodules. The female-to-male ratio was 15:1. Most of our patients were between 20 and 50 years old, with a mean age of 38.6 ± 11.6 years, distributed almost equally between 20 and 34 years (34.3%) and 35 and less than 50 years (36.5%). A family history of thyroid disorders was more frequent in the nodule group (*p* = 0.011), particularly for goiter or thyroid cancer (5.2% vs. 1.0%). Overweight and obesity were prevalent (~90%). BMI did not significantly differ between individuals with and without nodules (*p* = 0.728).

**Table 1:** Demographic and clinical characteristics of patients with Hashimoto’s thyroiditis.

Variable	Total (*n* = 408)	Nodules, yes (*n* = 96)	Nodules, no (*n* = 312)	*P*-value
Demographic characteristics				
Gender, female	383 (93.9%)	92 (95.8%)	291 (93.3%)	0.360
Age, years, mean ± SD	38.6 ± 11.6	42.5 ± 10.9	37.4 ± 11.6	<0.001*
Age category, years				0.001*
<20	26 (6.4%)	1 (1.0%)	25 (8.0%)	
20 to <35	140 (34.3%)	24 (25.0%)	116 (37.2%)	
35 to <50	149 (36.5%)	39 (40.6%)	110 (35.3%)	
≥50	93 (22.8%)	32 (33.3%)	61 (19.6%)	
Marital status				0.021*
Married	341 (83.6%)	89 (92.7%)	252 (80.8%)	
Single/divorced	67 (16.4%)	7 (7.3%)	60 (19.2%)	
Children number, median (IQR)	3.0 (1.0, 3.2)	3.0 (2.0, 4.0)	2.0 (1.0, 3.0)	0.010*
Height, cm, mean ± SD	160.9 ± 9.5	161.7 ± 9.5	160.6 ± 9.5	0.313
Weight, kg, mean ± SD	85.1 ± 20.8	86.4 ± 21.4	84.7 ± 20.6	0.463
BMI, kg/m², mean ± SD	32.8 ± 7.7	33.1 ± 8.1	32.8 ± 7.6	0.728
BMI category, kg/m²				0.152
Normal	39 (9.6%)	13 (13.5%)	26 (8.3%)	
Overweight	130 (31.9%)	26 (27.1%)	104 (33.3%)	
Obese	239 (58.6%)	57 (59.4%)	182 (58.4%)	
Family history				0.011*
No	319 (78.2%)	80 (82.5%)	239 (76.8%)	
Hypothyroidism	68 (16.7%)	9 (9.3%)	59 (19.0%)	
Hyperthyroidism	13 (3.2%)	3 (3.1%)	10 (3.2%)	
Goiter/cancer	8 (2.0%)	5 (5.2%)	3 (1.0%)	
Clinical characteristics				
Compressive manifestation, yes	90 (22.1%)	32 (33.0%)	58 (18.6%)	0.003*
Clinical picture				0.048*
Asymptomatic	54 (13.2%)	20 (20.6%)	34 (10.9%)	
Hypo-symptoms	319 (78.2%)	69 (71.1%)	250 (80.4%)	
Hyper-symptoms	35 (8.6%)	8 (8.2%)	27 (8.7%)	

Statistically significant at p < 0.05. Chi-square or Fisher’s exact tests were used for categorical data. An independent t-test was applied for normally distributed variables (mean ± SD), and the Mann–Whitney U test for skewed data (median, IQR). Compressive symptoms include dyspnea, dysphagia, neck swelling, neck pain, or hoarseness.

SD, standard deviation; IQR, interquartile range; BMI, body mass index; DTE, diffuse thyroid enlargement.

The symbol * means significant difference, p value < 0.05.

**Figure 1:**
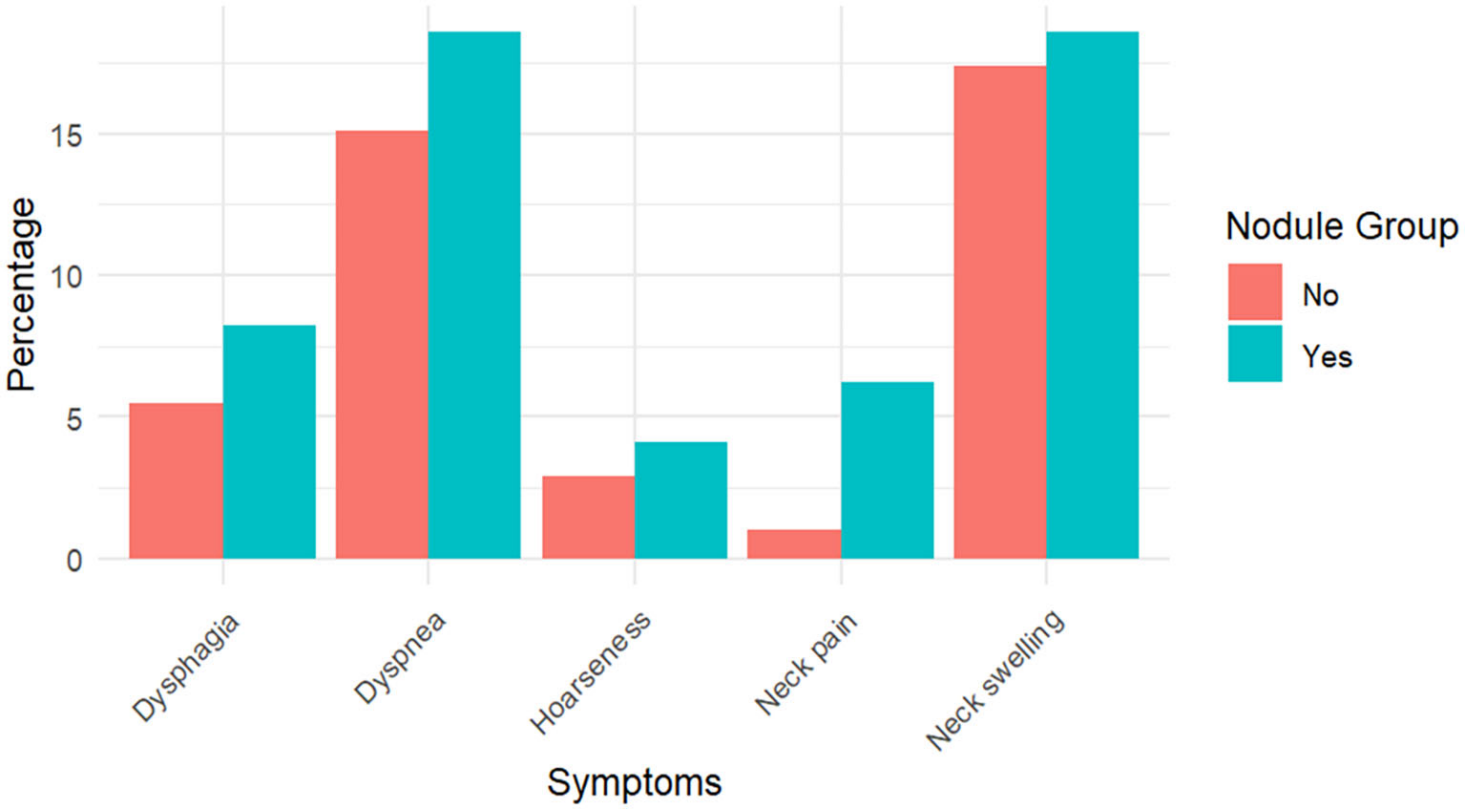
Frequency of compressive symptoms among participants with nodules (*N* = 96) and without nodules (*N* = 312). Values are presented as percentages with corresponding absolute numbers.

As shown in **Table 1**, the proportion of participants with compressive symptoms was higher in the nodule group (33.0% vs. 18.6%, *p* = 0.003). **Figure 1** further compares specific symptoms. Neck swelling was the most frequent symptom, reported by 18.6% (*n* = 18) of those with nodules and 17.4% (*n* = 54) without. Dysphagia occurred in 8.2% (*n* = 8) of the nodular group versus 5.5% (*n* = 17) of the non-nodular group. Dyspnea was reported in 18.6% (*n* = 18) compared to 15.1% (*n* = 47). Hoarseness was higher in the nodular group (4.1%, *n* = 4 vs. 2.9%, *n* = 9). Neck pain showed the most significant relative difference, being more common in participants with nodules (6.2%, *n* = 6 vs. 1.0%, *n* = 3; *p* = 0.003).

### Hypo- and hyperthyroid symptoms and signs among the study population

**Figure 2:**
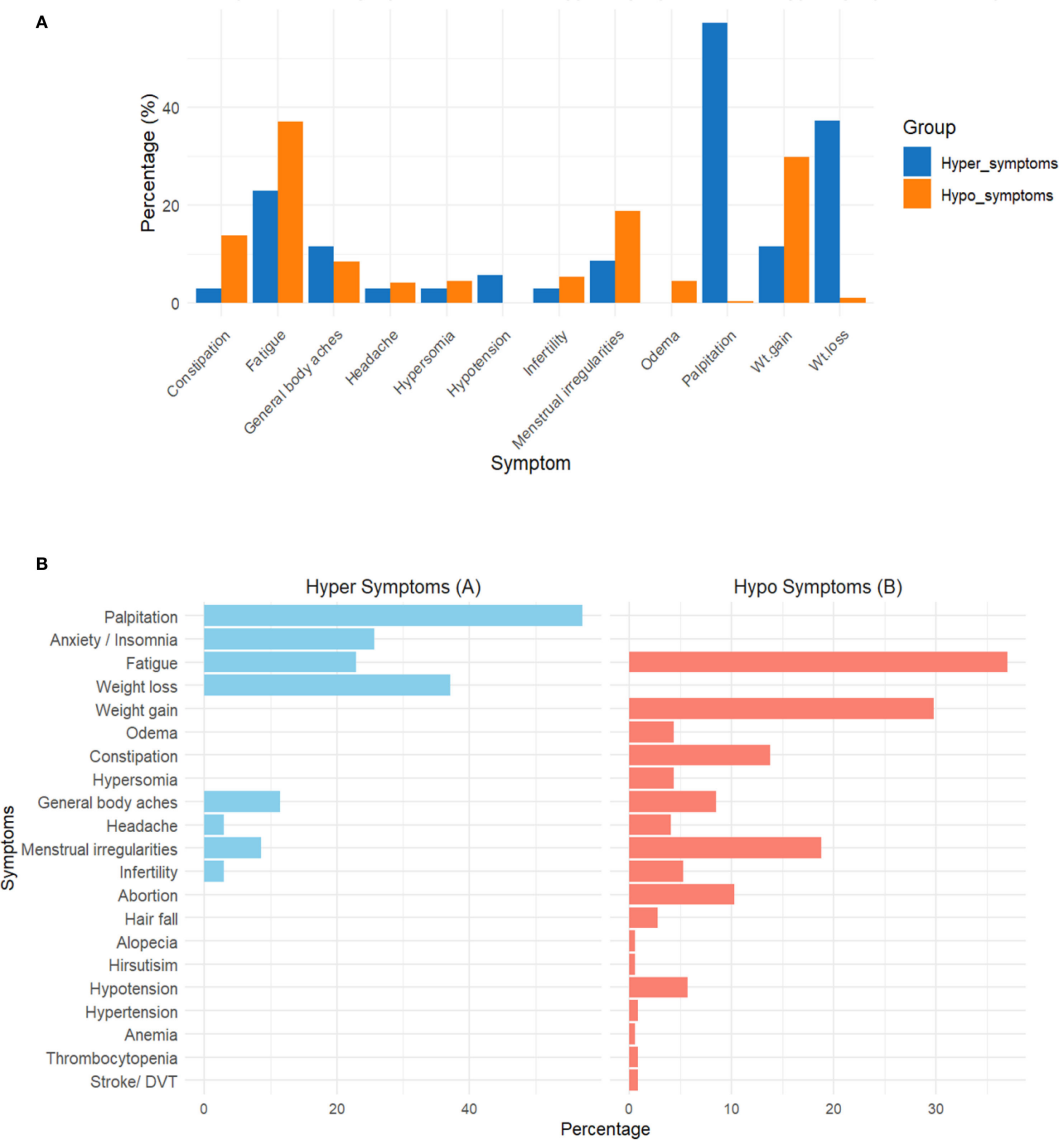
Distribution of clinical symptoms among participants with hyperthyroidism **(A)** and hypothyroidism **(B)**. Symptoms were categorized based on clinical presentation reported at enrollment. Data are presented as percentages of affected participants. *This figure is intended for descriptive comparison only; no statistical testing was performed between the two groups.

**Figure 2** illustrates the proportion of various hypo- and hyperthyroid symptoms. Fatigue was the most common symptom in patients with hypothyroid manifestations (37.0%), while palpitations were the most frequent symptom in hyperthyroid manifestations (57.1%). Weight gain was observed in 29.8% of hypothyroid cases, whereas weight loss was observed in 37.1% of hyperthyroid cases. Notably, neck swelling was significantly more common in patients showing manifestations of hypothyroidism (20.7%) compared to those with hyperthyroidism (5.7%). Menstrual irregularities occur in both hypothyroidism (18.8%) and hyperthyroidism (8.6%). Hypothyroidism was also associated with constipation (13.8%), edema (4.4%), somnolence (4.4%), hair fall (2.8%), and hypotension (5.7%).

### Thyroid function tests of the study population

Subclinical hypothyroidism was the most common presentation (48.8%, 199/408), followed by overt hypothyroidism (32.1%, 131/408) and euthyroidism (13.4%, 54/408). Overt and subclinical hyperthyroidism were the least commonly encountered, with 3.4% (14/408) and 2.4% (10/408), respectively. Lower median TSH levels were found in the nodular group (6.5 vs. 8.9 mIU/L, *p* < 0.001) as well as higher FT3 (3.1 vs. 2.9 pg/mL, *p* = 0.006) and FT4 (1.1 vs. 1.1 ng/dL, *p* = 0.008) levels. Overt hypothyroidism was less frequent in the nodule group (15.6% vs. 37.2%), whereas euthyroidism was more common (20.8% vs. 10.9%, *p* < 0.001) (**Table 2**).

**Table 2:** Laboratory and ultrasonographic characteristics of patients with Hashimoto’s thyroiditis.

Variable	Total (*n* = 408)	Nodules, yes (*n* = 96)	Nodules, no (*n* = 312)	*P*-value
Laboratory characteristics				
TSH (mIU/L), median (IQR)	8.4 (5.3, 15.6)	6.5 (3.0, 10.7)	8.9 (5.9, 18.6)	<0.001
FT3 (pg/mL), median (IQR)	2.9 (2.6, 3.3)	3.1 (2.7, 3.5)	2.9 (2.5, 3.2)	0.006
FT4 (ng/dL), median (IQR)	1.1 (0.9, 1.2)	1.1 (1.0, 1.3)	1.1 (0.8, 1.2)	0.008
FT3/FT4, median (IQR)	2.7 (2.3, 3.4)	2.7 (2.4, 3.4)	2.7 (2.3, 3.3)	0.835
TPOAbs (IU/mL), median (IQR)	140.2 (67.0, 417.0)	78.7 (17.0, 238.9)	190.0 (78.7, 431.9)	<0.001
TgAb (IU/mL), median (IQR)	90.8 (20.0, 330.2)	50.0 (17.1, 191.8)	107.5 (21.0, 378.2)	0.014
Cholesterol (mg/dL), median (IQR)	173.0 (144.0, 203.2)	177.5 (144.0, 197.2)	170.5 (144.0, 204.0)	0.861
LDL-C (mg/dL), median (IQR)	105.0 (91.8, 121.0)	109.0 (99.0, 121.5)	102.5 (89.0, 121.0)	0.058
HDL-C (mg/dL), median (IQR)	45.0 (39.0, 52.0)	45.0 (40.6, 54.0)	45.0 (39.0, 52.0)	0.181
TPOAbs, positive	329 (80.6%)	59 (61.5%)	270 (86.5%)	<0.001*
Anti-TgAb, positive	187 (45.8%)	35 (36.5%)	152 (48.7%)	0.035
Laboratory diagnosis				<0.001*
Euthyroidism	54 (13.2%)	20 (20.8%)	34 (10.9%)	
Overt hyperthyroidism	14 (3.4%)	4 (4.2%)	10 (3.2%)	
Overt hypothyroidism	131 (32.1%)	15 (15.6%)	116 (37.2%)	
Subclinical hyperthyroidism	10 (2.5%)	5 (5.2%)	5 (1.6%)	
Subclinical hypothyroidism	199 (48.8%)	52 (54.2%)	147 (47.1%)	
Ultrasound characteristics				
TGV (mL), median (IQR)	15.6 (10.9, 21.7)	16.2 (11.6, 21.9)	15.2 (10.8, 21.6)	0.328
DTE, yes	146 (35.8%)	36 (37.5%)	110 (35.3%)	0.688
Pseudo-nodularity, yes	246 (60.3%)	53 (55.2%)	193 (61.9%)	0.244
Echotexture, heterogeneous	388 (95.1%)	91 (94.8%)	297 (95.2%)	0.874
Echogenicity grades				0.248
1	108 (26.5%)	29 (30.2%)	79 (25.3%)	
2	198 (48.5%)	49 (51.0%)	149 (47.8%)	
3	102 (25.0%)	18 (18.8%)	84 (26.9%)	
Vascularity				0.659
No	22 (5.4%)	3 (3.1%)	19 (6.1%)	
Mild	313 (76.7%)	77 (80.2%)	236 (75.6%)	
Moderate	67 (16.4%)	15 (15.6%)	52 (16.7%)	
Marked	6 (1.5%)	1 (1.0%)	5 (1.6%)	

Statistically significant at p < 0.05. Chi-square or Fisher’s exact tests were used for categorical data. An independent t-test was applied for normally distributed variables (mean ± SD), and the Mann–Whitney U test for skewed data (median, IQR).

SD, standard deviation; IQR, interquartile range; TSH, thyroid-stimulating hormone; FT3, free triiodothyronine; FT4, free thyroxine; TPOAbs, anti-thyroid peroxidase antibodies; TgAb, anti-thyroglobulin antibodies; LDL-C, low-density lipoprotein cholesterol; HDL-C, high-density lipoprotein cholesterol; TGV, thyroid gland volume; DTE, diffuse thyroid enlargement.

* indicates statistical significance at p < 0.05.

### Thyroid antibody profile of the study population

Overall, 81% and 46% of the samples tested positive for TPOAb and TgAb, respectively, and 12.5% were seronegative. Seropositivity was significantly higher in the non-nodular group (87% vs. 61% for TPOAb and 47% vs. 35% for TgAb). TPOAb (78.7 vs. 190.0 IU/mL, *p* < 0.001) and TgAb (50.0 vs. 107.5 IU/mL, *p* = 0.014) levels were lower in individuals with nodules, along with reduced positivity rates for both antibodies (**Table 2**).

### Ultrasound findings of the study population

Median TGV was 15.6 mL (IQR: 10.9–21.7), with diffuse thyroid enlargement (DTE) in 35.8%, pseudo-nodularity in 60.3%, heterogeneous echotexture in 95.1%, and mild increase in vascularity in 76.7%. None of these parameters showed a significant difference between the nodular and non-nodular groups (**Table 2**).

### Sonographic, cytologic, and histopathological features in patients with nodular presentation

**Table 3** reveals that among the nodular group (*n* = 96), 68.8% had a solitary thyroid nodule and 31.2% had multinodular goiter. A total of 133 nodules were identified: solid (68.4%), mixed cystic-solid (21.1%), pure cystic (6%), and spongiform (4.5%). Hypoechogenicity (45.9%) and isoechogenicity (39.1%) were the most common, while fewer nodules were hyperechoic (10.5%) or anechoic (4.5%). Well-defined borders were observed in 76.6% of cases, and regular margins in 91%. Most nodules were wider than tall (94.7%), with a small percentage being taller than wide (5.3%). There were 86.5% of nodules that had no calcifications, and macrocalcifications (7.5%), rim/eggshell calcifications (1.5%), and microcalcifications (4.5%) were less frequent. A total of 41.3% of nodules showed no vascularity, 33.1% showed peripheral vascularity, and 25.6% showed central/mixed vascularity. Using the TIRADS classification, most nodules were classified as TIRADS 3 (38.4%) and TIRADS 4 (32.3%), with fewer categorized as TIRADS 5 (8.3%), TIRADS 2 (12%), or TIRADS 1 (9%).

**Table 3:** Sonographic, cytological, and histopathological characteristics in patients with nodular presentation.

Variable	Number (%)
Nodular Hashimoto’s thyroiditis (*n* = 96)	
• Solitary thyroid nodule	66 (68.8)
• Multinodular goiter	30 (31.2)
Total nodules (*n* = 133)	
Mean thyroid nodule diameter (cm), mean ± SD	1.47 ± 0.79
Characteristics of the nodules (*n* = 133)	
Composition (*n* = 133 nodules)	
– Solid	91 (68.4)
– Cystic	8 (6.0)
– Mixed	28 (21.1)
– Spongiform	6 (4.5)
Echogenicity (*n* = 133 nodules)	
– Anechoic	6 (4.5)
– Hyperechoic	14 (10.5)
– Isoechoic	52 (39.1)
– Hypoechoic	61 (45.9)
Border (*n* = 133 nodules)	
– Well-defined	102 (76.7)
– Ill-defined	31 (23.3)
Margin (*n* = 133 nodules)	
– Regular	121 (91.0)
– Irregular	12 (9.0)
Growth pattern (*n* = 133 nodules)	
– Wide > tall	126 (94.7)
– Tall > wide	7 (5.3)
Calcifications (*n* = 133 nodules)	
– None	115 (86.5)
– Macrocalcification	10 (7.5)
– Rim/eggshell	2 (1.5)
– Microcalcification	6 (4.5)
Vascularity (*n* = 133 nodules)	
– None	55 (41.3)
– Peripheral	44 (33.1)
– Central/mixed	34 (25.6)
TIRADS score (*n* = 133 nodules)	
– TIRADS-1	12 (9.0)
– TIRADS-2	16 (12.0)
– TIRADS-3	51 (38.4)
– TIRADS-4	43 (32.3)
– TIRADS-5	11 (8.3)
FNAC (*n* = 49)	
– Non-diagnostic (Bethesda I)	2 (4.1)
– Benign (Bethesda II)	31 (63.2)
– Indeterminate (Bethesda III–V)	16 (32.7)
– Malignant (Bethesda VI)	0 (0.0)
Postoperative histopathology (*n* = 18)	
Benign	10 (55.6)
• Thyroiditis	3 (30.0)
• Adenomatous goiter	6 (60.0)
• Hürthle cell adenoma	1 (10.0)
Malignant (papillary thyroid carcinoma)	8 (44.4)
• Microcarcinoma	1 (12.5)
• Classic variant	3 (37.5)
• Multifocal	2 (25.0)
• FVPTC	2 (25.0)

Data are presented as numbers (%), unless otherwise specified. For nodules, the denominators were as follows: total patients with nodular HT (n = 96), total nodules (n = 133), FNAC nodules (n = 49), and operated cases (n = 18). Percentages are calculated according to the respective denominators. Statistical significance was considered at p < 0.05.

FNAC, fine−needle aspiration cytology; FVPTC, follicular variant of papillary thyroid carcinoma; TIRADS, Thyroid Imaging Reporting and Data System; SD, standard deviation.

FNAC was performed on 43 patients with 49 nodules, showing 63.2% (Bethesda II), 32.7% indeterminate (Bethesda III–V; 3 cases Bethesda III, 11 cases Bethesda IV, and 2 cases Bethesda V), and 4.1% non-diagnostic (Bethesda I), with no malignant cases (Bethesda VI). Postoperative histopathology (*n* = 18) identified benign pathology in 10/18 (55.6%), including adenomatous goiter (60%), Hashimoto’s thyroiditis (30%), and Hürthle cell adenoma (10%). Thyroid cancer was present in 8/18 (44.4%), all diagnosed as papillary thyroid cancer; 12.5% were microcarcinomas, 37.5% classic variants, 25% multifocal, and 25% follicular variant papillary thyroid carcinoma (FVPTC) (**Table 3**).

Notably, higher median TSH levels were observed in patients with malignant nodules compared to those with benign nodules, as indicated by postoperative histopathology (9 vs. 6 mIU/L). Additionally, median TPOAbs and anti-TgAb levels were higher in malignant cases (172 and 821 IU/L, respectively) than in benign cases (65.6 and 170 IU/L, respectively).

### Concordance between preoperative FNAC and postoperative histopathology in the 18 operated HT cases

Overall, FNAC demonstrated an 88.9% concordance with final histopathology (16/18 cases). Bethesda II and V showed complete agreement with benign and malignant outcomes, respectively. Bethesda III revealed indeterminate results (two benign, one malignant, 33.33% malignancy rate). Bethesda IV cases had a malignancy rate of 45.5%, slightly higher than the expected risk of malignancy set by the BSTRC upper expected risk range for this category. The detailed concordance of FNAC and postoperative histopathology is attached as **Supplementary Table S1**.

### Benign and malignant patients’ characteristics

#### Malignant cases

Malignant cases (*n* = 8) were categorized according to BSTRC as follows: one case with Bethesda III, five cases with Bethesda IV, and two cases with Bethesda V. Seventy-five percent had a TIRADS score of 5, and 25% had a TIRADS score of 4. High-resolution ultrasound (HRUS) showed 100% solid and hypoechoic nodules; 62.5% were ill-defined, 75% were irregular, and 75% harbored microcalcifications (8.3%, 8/96 of the total cohort and 1.96%, 8/408 of the nodular group had postoperative malignant histopathology).

### Benign cases

Thirty-five patients were confirmed to have benign conditions based on FNAC and postoperative histopathology; 10 out of 35 patients were identified with nodular Hashimoto’s thyroiditis. Eight cases were diagnosed by FNAC (Bethesda II), and two by postoperative histology (Bethesda IV). One FNAC-diagnosed case underwent surgery due to compressive symptoms and was confirmed by postoperative histology as lymphocytic thyroiditis. Seventy-five percent of nodular HTs were solid, while 25% were mixed cystic-solid. Fifty percent were hypoechoic, 25% were isoechoic, and 25% were hyperechoic. Seventy-five percent had ill-defined borders, 25% had irregular borders, and 12.5% exhibited taller-than-wide growth. None contained calcifications. TIRADS scores were 3 (*n* = 3), 4 (*n* = 5), and 5 (*n* = 2).

#### Benign nodules other than nodular Hashimoto’s thyroiditis

The remaining 25 patients were diagnosed through FNAC or postoperative histopathology (19 with Bethesda II, 2 with Bethesda III, and 4 with Bethesda IV). Seventy-one percent were solid, and 29% were mixed cystic-solid; 54.8% were hypoechoic, and 45.2% were isoechoic; 61.3% had well-defined borders, 80.6% had regular margins, and 35.4% showed evidence of calcifications, including macrocalcifications and eggshell calcifications; 6.4% had a TIRADS score of 2, 41.9% scored 3, 48.4% scored 4, and 3.2% scored 5.

### Predictors of presentation with nodules in HT patients

Individuals aged 35–50 years had seven times higher probability to be presented with nodules (OR = 7.023, 95% CI: 1.447–34.090, *p* = 0.016), while those aged ≥50 years had more than eight times higher odds (OR = 8.589, 95% CI: 1.740–42.402, *p* = 0.008) compared to individuals under 20. A family history of goiter or thyroid cancer was linked to a fivefold increase in odds (OR = 5.177, 95% CI: 1.055–25.403, *p* = 0.043). Among thyroid-related biomarkers, lower TSH levels were associated with increased odds of nodules (OR = 0.981, 95% CI: 0.966–0.997, *p* = 0.017), and TPOAb levels also decreased the odds (OR = 0.998, 95% CI: 0.997–0.999, *p* = 0.004). However, an increase in TGV was associated with a 36% increase in the probability of having nodules (OR = 1.036, 95% CI: 1.012–1.060, *p* = 0.003). Model diagnostics indicated an acceptable fit (Hosmer–Lemeshow *p* = 0.233; AIC = 415.63; McFadden’s *R*² = 0.143; Nagelkerke’s *R*² = 0.218), with no evidence of multicollinearity (all VIF < 2). Thus, this supports the reliability of our findings, despite the small nodular subgroup and wide confidence intervals for some estimates.

## Discussion

There was a clear female predominance among patients with HT in this study, with female respondents accounting for 93.9% and male respondents 6.1% (a ratio of 15:1), which is higher than the commonly reported 4–8:1 female-to-male ratio. Age range was equally distributed between the 20–34- (34.3%) and 35–50-year (36.5%) age groups. Similarly, Erdogan et al. (14) reported a high female predominance with age, with most of their population clustered between 30 and 50 years old. Almahari et al. (15) reported a slightly higher age predominance of patients with HT, specifically between 41 and 60 years. The observed female predominance of patients with Hashimoto’s thyroiditis is likely related to the X chromosome, which contains multiple sex- and immune-related genes crucial for preserving immune tolerance (16).

Our analysis found that the mean age of patients with thyroid nodules was significantly higher than that of the non-nodular group, although no gender differences were elicited. Consistent with our findings, a large-scale study in Korea showed a higher prevalence of thyroid nodules detected by US in women and older age groups, with a notable increase in the size of thyroid nodules associated with these two variables (17).

**Figure 3:**
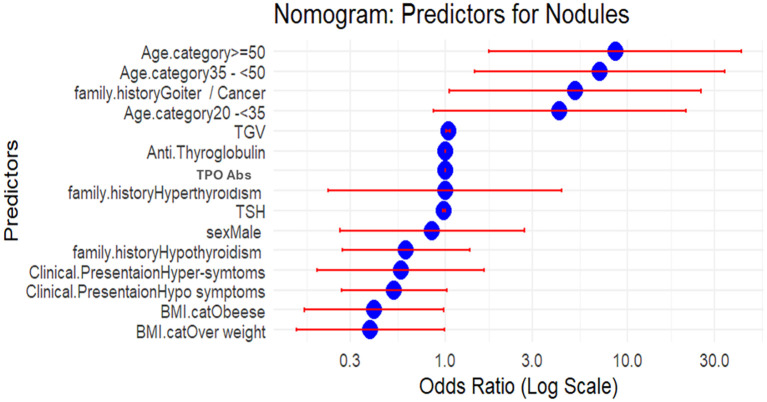
Nomogram illustrating the odds ratios (ORs) of independent predictors for nodular presentation in Hashimoto’s thyroiditis (*n* = 408). ORs were derived from multivariable logistic regression, with the *x*-axis presented on a logarithmic scale. An OR greater than 1 indicates increased relative risk of nodularity, whereas an OR less than 1 reflects a protective effect. BMI, body mass index; TSH, thyroid-stimulating hormone; FT3, free triiodothyronine; FT4, free tetraiodothyronine; TPOAbs, thyroperoxidase antibodies; TGV, total glandular volume; DTE, diffuse thyroid enlargement. Compressive manifestation: dyspnea, dysphagia, neck swelling, neck pain, or hoarseness.

A positive family history of AITDs was present in 22% of cases (89 out of 408). Familial studies strongly support the genetic basis of HT, where shared environmental factors may contribute to familial clustering among relatives. A family history of goiter and/or cancer was identified in eight cases within our studied cohort, mainly in the nodular group. In line with our findings, a cross-sectional study in Iran found that a family history of thyroid disorders and goiter, along with female sex and aging, was associated with an increased risk of thyroid nodules (18) (**Figure 3**, **Table 4**).

**Table 4:** Predictors of nodular presentation among HT patients (multivariate logistic regression, *N* = 408; nodular cases = 96).

Variable	OR	95% CI	*P*-value
Age category (ref: <20 years)			
20–<35 years	4.236	0.859–20.904	0.076
35–<50 years	7.023	1.447–34.090	0.016*
≥50 years	8.589	1.740–42.402	0.008**
Sex (ref: female)			
Male	0.847	0.263–2.728	0.781
BMI category (ref: normal weight)			
Obese	0.406	0.168–0.982	0.045*
Overweight	0.388	0.152–0.992	0.048*
Family history (ref: none)			
Goiter/thyroid cancer	5.177	1.055–25.403	0.043*
Hyperthyroidism	0.996	0.228–4.361	0.996
Hypothyroidism	0.607	0.271–1.360	0.225
Clinical presentation (ref: asymptomatic)			
Hyperthyroid symptoms	0.569	0.199–1.628	0.293
Hypothyroid symptoms	0.524	0.269–1.023	0.058
Biomarkers (continuous)			
TSH (mIU/L)	0.981	0.966–0.997	0.017*
TPOAbs (IU/mL)	0.998	0.997–0.999	0.004**
Anti-thyroglobulin (IU/mL)	0.999	0.999–1.002	0.354
TGV (mL)	1.036	1.012–1.060	0.003**

Model diagnostics:

Hosmer–Lemeshow goodness-of-fit test: *p* = 0.233.

Null deviance = 447.55, *df* = 407.

AIC = 415.63.

McFadden’s *R*² = 0.1428.

Nagelkerke’s *R*² = 0.2177.

All VIF < 2 (no multicollinearity).

Significance: *p* < 0.05 (), *p* < 0.01 (**).

CI, confidence interval; BMI, body mass index; TSH, thyroid-stimulating hormone; FT3, free triiodothyronine; FT4, free tetraiodothyronine; TPOAbs, thyroperoxidase antibodies; TgAb, anti-thyroglobulin antibodies; TGV, total glandular volume.

The symbol * means significant difference, p value < 0.05.

Most patients in our sample were either overweight or obese, with a mean BMI of 32.8 ± 7.7 kg/m². There was no significant difference in BMI between the nodular and non-nodular groups. Research, including a meta-analysis by Rong SH, has highlighted that obesity is associated with increased odds of developing thyroid autoimmunity, elevated TSH, and hypothyroidism. Hyperleptinemia observed in obesity was found to be associated with an augmented production of cytokines that perpetuate cellular immune responses by enhancing the function of T helper-1 cells and suppressing T-regulatory cells (19). Furthermore, obesity has been linked to higher thyroid volume and an increased risk of thyroid nodules and cancer, likely due to chronic low-grade inflammation, hyperlipidemia, hyperestrogenemia, and hyperinsulinemia, all of which may promote thyroid cell growth and differentiation (20).

HT presents with a wide range of manifestations in clinical practice, from positive circulating thyroid autoantibodies with euthyroid status up to life-threatening complications. A transient phase of thyrotoxicosis may occur initially, typically resolving spontaneously (21). In our cohort, nearly one in five patients reported compressive symptoms, which were more common among those with thyroid nodules. Symptoms of hypothyroidism were most prevalent, with fatigue being the chief complaint, followed by weight gain, menstrual irregularities in female respondents, constipation, somnolence, generalized edema, and hair loss. Hyperthyroid symptoms were reported by 8.6% of the patients. Thyroid function tests confirmed that 81% of patients had hypothyroidism, both overt and subclinical, with the latter being more common. Notably, patients with nodular presentations had a lower average serum TSH level compared to those without nodules, while those with malignant cytology had higher TSH than those with benign cytology.

The high prevalence of hypothyroidism, as shown by both clinical and biochemical data, is likely due to the inclusion of newly diagnosed, untreated cases. In a study in India, 114 patients with cytologically proven HT were found to be either asymptomatic or experiencing non-specific symptoms, such as easy fatigue, with tenderness of the thyroid being the second most common symptom. However, in agreement with our findings, most of the patients in their study had subclinical hypothyroidism based on biochemical confirmation (22).

In our study, nearly 81% of our samples were positive for TPOAb, and 49% were positive for TgAb. It has been observed that TgAb appears before the emergence of TPOAb in thyroiditis. However, TPOAbs are more directly involved in thyrocyte damage through antibody-dependent cell cytotoxicity (23). Some studies suggest that TPOAbs are markers of progressive thyroid failure rather than direct pathogenic factors (24). Seronegativity to both studied autoantibodies was observed in 12.5% of cases after excluding all potential confounding factors. Rotondi et al. (6) compared seronegative and classical seropositive HT cases, highlighting that patients with seronegative disease show a milder form of thyroid failure, evidenced by lower thyroxine replacement doses needed to attain euthyroidism, probably reflecting a weaker genetic predisposition. Additionally, the risk of developing nodular disease was similar in both groups. Baker JR et al. suggested that seronegative patients may have thyroid autoantibodies confined within the thyroid gland, rather than being detectable in the serum (25). In contrast to our findings, Erdogan M. et al. (14) observed higher positivity rates: 92% of women and 93.2% of men for TgAb and 98.4% of women and 100% of men for TPOAb. Croce et al. (11) reported a 79% positivity rate for antithyroid antibodies among patients with autoimmune thyroiditis. Interestingly, they found that antibody status independently predicted the required dose of thyroxine replacement in these individuals. In our study, lower antibody positivity rates were observed in patients with nodular presentation; however, higher titers of thyroid autoantibodies were associated with an increased risk of thyroid cancer in patients with thyroid nodules. Overall, antibody positivity did not affect the risk of developing thyroid nodules.

Ultrasonography plays a key role in patients with HT, especially in seronegative cases. US allows for an accurate assessment of volume and echogenicity and can detect non-palpable nodules. Typical findings include heterogeneous parenchymal echotexture, echogenic septations, micronodularity, and hypervascularity. The wide availability of the US, being inexpensive and non-invasive, makes it a valuable tool in clinical practice.

Diffuse hypoechogenicity of thyroid parenchyma was observed in all enrolled cases, with 95% showing a diffuse, heterogeneous echotexture and 60% showing pseudo-nodularity. Diffuse thyroid enlargement was observed in 35.8% of cases, with a higher mean total thyroid volume in the nodular group, although this difference was not statistically significant. Three-quarters of the sample displayed a slight increase in vascularity. No significant difference in ultrasound findings was noted between the nodular and non-nodular groups, except for the presence of discrete thyroid nodules.

Thyroid nodules, both benign and malignant, are commonly observed in patients with HT. Malignant nodules are often identified as papillary thyroid cancer or thyroid lymphoma (8, 26). It is essential to distinguish between true thyroid nodules and those presenting with a focal form of thyroiditis (nodular HT). Nodular HT has a variable presentation, with an estimated prevalence of 5% among biopsied nodules (15, 27). The final diagnosis relies on cytopathology, which plays an incremental role in differentiating benign and suspicious nodules. This helps to avoid unnecessary surgeries in most cases (7, 26). Erdogan et al. (14), based on thyroid ultrasonography, reported normal findings in 12.9%, diffuse enlargement in 23.6%, single nodule in 52.2%, and multiple nodules in 11.3% of female patients; in male patients, 12% had normal findings, 36% had diffuse enlargement, 32% had single nodule, and 20% had multiple nodules. Almost 27% of the patients with nodules in their study underwent FNAC, resulting in benign cytology in 95% of cases, 28% with nodular Hashimoto, and 65% with benign nodular findings.

In our cohort, 23.5% had US evidence of thyroid nodules. Multinodular goiter (MNG) was present in 31.6%, and 68.4% had solitary thyroid nodules. Most nodules were solid, hypoechoic, and lacked calcifications; approximately a quarter had ill-defined borders. Using the ACR TIRADS scoring system, most nodules were classified as TIRADS 3 and TIRADS 4. FNAC was performed on 36.8% of the nodules, revealing benign cytology based on BSTRC in 63.2% and indeterminate cytology in 32.7%. Papillary thyroid cancer was identified in 8% of the patients with nodules and 2% of the total HT cohort. Isik et al. (28) found that nearly a quarter of cases with HT had thyroid nodules, with solid nodules in 91%, hypoechoic nodules in 38.4%, microcalcifications in 17.8%, and irregular margins in 4.1%. FNAC results showed 75% benign, 6.7% indeterminate, and 2.7% malignant. Thyroid cancer was found in 1.96% of HT cases in our study and in 8.3% of the nodular group in their study.

Chronic inflammation, the trophic effects of high TSH, and elevated thyroid autoantibodies are the proposed mechanisms linking HT and thyroid cancer, especially papillary thyroid cancer (29). The coexistence of HT and PTC has been documented in various studies. An Italian study noted a significant prevalence of HT and PTC, particularly its nodular variant, compared to the diffuse form of HT (30), emphasizing the need for meticulous evaluation of patients with HT, especially those with nodules. North American studies and the Surveillance, Epidemiology, and End Results (SEER) database also highlighted an elevated risk of PTC in patients with HT, although it is generally associated with favorable outcomes (31). A recent meta-analysis conducted by Ma C. et al. (32) found concurrent HT in one-third of the PTC patients. These patients tend to be younger, are more often women, have lower rates of lymph node metastasis and capsular invasion, and have a lower incidence of BRAF mutations. However, we were unable to obtain the detailed clinicopathological criteria of the malignant cases with HT, as the total number of malignant cases with HT (2% of the total cohort studied) was too small to allow for a meaningful statistical comparison.

HT is also associated with primary thyroid lymphoma, which typically presents as a large, solid hypoechoic to markedly hypoechoic mass on ultrasound, characterized by internal vascularity with no evidence of calcification, as described by Sharma A et al. (33).

Although implementing the usual criteria for distinguishing benign from malignant nodules based on HRUS has reasonable reproducibility, distinguishing between benign and malignant thyroid nodules in the setting of HT remains challenging. This difficulty arises from the variable sonographic features of benign nodules, including cases of nodular HT. Researchers have observed that the sonographic appearance of nodular HT can present in different forms, ranging from solid to mixed solid and cystic, with hypoechoic or hyperechoic characteristics, and ill-defined margins (7, 26, 27). Oppenheimer et al. (27) found that the most common sonographic form of nodular HT was a solid, hypoechoic nodule with a thin peripheral halo. Similarly, in our study, nodular HT exhibited diverse US features, ranging from benign to suspicious, as reflected by a TIRADS score of 3 to 5. In agreement with our findings, Almahari et al. (15) reported that nearly 17.7% of the biopsied thyroid nodules in cases of HT were cytologically diagnosed as nodular HT, which closely matches our figure of 20.4%. Andersen et al. (26) highlighted that margin distinction and vascularity overlap in both benign and malignant nodules, likely attributed to the high vascularity of the thyroid gland in HT. Furthermore, they emphasized that all types of calcifications, particularly microcalcifications and tiny bright reflectors, can be found in malignant nodules in the setting of HT. These findings were consistent with ours, where we observed a high TIRADS score (75% TIRADS-5 and 25% TIRADS-4) with microcalcifications in 62.5% of PTC cases. Thus, high TIRADS scores, mainly with microcalcifications, were associated with increased malignancy risk, where microcalcifications serve as the most sensitive marker for differentiating between nodular HT and PTC. Despite this, experts recommend the development of specific US models tailored for HT with nodules to avoid unnecessary biopsies (15, 26).

We constructed a model to assess the factors associated with nodular presentation in cases with HT. Older age, family history of goiter or cancer, and increased thyroid volume are associated with a higher risk of nodules. Gender and obesity are not considered risk factors in our study, possibly due to the higher prevalence of overweight and obesity in both groups. Although patients with malignant nodules had higher TSH levels, the multivariate regression identified lower TSH as an independent, though weak, predictor (OR close to 1) for nodular disease. This observation should be interpreted cautiously, as TSH is linked to thyrocyte growth and differentiation and high levels are associated with increased cancer risk. Several studies may support our findings. Gitti et al. (34) found that low TSH levels were significantly correlated with TIRADS 4 nodules, which are typically benign. Additionally, Li et al. identified low TSH levels (0.27–1.41 IU/mL) as a risk factor for thyroid nodules, suggesting that TSH variation within the normal range can influence the development of nodules (35). Other influencing factors, such as hyperinsulinemia and obesity (19, 20, 36), may interact with TSH in complex ways that are not captured in isolated analysis. Moreover, TSH variability must be considered: A single TSH measurement may not accurately reflect long-term levels that influence nodule development. Additionally, we did not find an interaction between TSH and TPOAb in our study.

Our results are consistent with Lima PC et al. (37), who found that a larger thyroid volume increased the likelihood of thyroid nodules in both Graves’ disease and HT. However, they observed that older age predicted the development of thyroid nodules in patients with GD, but not in those with HT. Kalkan et al. (36) discovered that patients with HT and thyroid nodules had lower TSH levels than those without nodules, supporting our findings.

### Strengths and limitations

Our study’s strengths include its cross-sectional design and consistent interpretation by one sonographer and one cytopathologist, which reduces interobserver bias. Limitations include a single-center study design, possible sampling bias, and reliance on hormonal and radiological diagnoses in seronegative cases. We attempted to mitigate this issue by excluding all confounding factors that might have elevated TSH levels and by repeating the thyroid profile. The small number of patients with HT and thyroid cancer limited statistical analysis of malignant cases. The inclusion of BMI in the regression model may be a minor limitation due to the exclusion of obesity in seronegative cases, which could potentially introduce a slight selection bias; however, 87.5% of cases were seropositive. The small number of the nodular group (*n* = 96) resulted in wide confidence intervals for some predictors, which may lead to potential overfitting. However, the model diagnostics indicated an adequate fit and no collinearity. Thus, the findings remain clinically meaningful, offering valuable insights into factors associated with nodule development in HT and emphasizing the need for validation in larger, multicenter studies to confirm their generalizability.

### Summary and conclusions

In summary, our cohort demonstrated strong female predominance and higher prevalence among individuals aged 20–50 years, consistent with the literature. Clinical presentation was variable, with compressive symptoms more common in patients with thyroid nodules. Subclinical hypothyroidism was the most frequent biochemical abnormality. Most patients were positive for thyroid autoantibodies, but 12.5% were seronegative, highlighting the importance of ultrasonographic evaluation. Diffuse thyroid enlargement was seen in about one-third of patients, and one-fifth had thyroid nodules. Accurate radiological assessment and FNAC are essential for managing nodules in HT, preventing unnecessary surgery. Older age, higher thyroid volume, and family history of goiter or cancer were major predictors of nodular presentation. Although patients with malignant nodules had higher TSH levels, the multivariate regression analysis indicated that lower TSH levels predicted nodular presentation overall. This likely reflects the complex interaction between TSH, autoimmunity, and thyroid growth dynamics in HT, as reported in prior studies. The malignancy rate in HT with nodules was 8% (1.96% of the total cohort), with microcalcifications strongly associated with malignancy.

### Recommendations

We recommend that patients diagnosed with HT be closely monitored through regular assessments of TSH and autoantibody titers, with particular attention to TSH levels. Patients with nodular disease should additionally undergo both clinical and cytological evaluations of the nodular lesions. Furthermore, further research is warranted to explore the genetic and environmental determinants of thyroid nodules, as well as to evaluate the efficacy of novel diagnostic and therapeutic approaches. Moreover, we suggest including patients with varying BMI to clarify the role of obesity in the development of thyroid nodules in individuals with HT. Comparative studies are needed to evaluate the relationship between TSH, thyroid autoantibodies, and nodular presentation in the setting of HT.

## Data Availability

The original contributions presented in the study are included in the article/**Supplementary Material**. Further inquiries can be directed to the corresponding author.
