# Supplementary material for: Hashimoto’s thyroiditis in an Egyptian cohort: clinical, functional, and ultrasonographic features with insights into nodule risk
Source: Front Endocrinol (Lausanne). 2025 Nov 6;16:1664047. doi: 10.3389/fendo.2025.1664047 (PMC12635913; doi:10.3389/fendo.2025.1664047)
Supplement: Supplementary file 1 [file Table1.docx]

**Correlation between FNAC(BSTRC), and postoperative histopathology in operated cases with HT (N=8)**

| FNAC (BSRTC) | Thyroiditis | Adenomatous goiter | Hürthle cell adenoma | PTC micro | PTC classic | FVPTC | Multifocal PTC | Total | Benign outcome (n) | Malignant outcome (n) | Concordance (benign vs malignant) |
| --- | --- | --- | --- | --- | --- | --- | --- | --- | --- | --- | --- |
| BII (Benign) | 1 | 1 | – | – | – | – | – | 2 | 2 | 0 | 100% concordant (benign) |
| BIII (AUS/FLUS) | – | 2 | – | – | – | – | 1 | 3 | 2 | 1 | 66.7% concordant (1 discordant) |
| BIV (Suspicious for follicular neoplasm) | 2 | 3 | 1 | 1 | 2 | 2 | – | 11 | 6 | 5 | 100% concordant (benign vs malignant) |
| BV (Suspicious for malignancy) | – | – | – | – | 1 | – | 1 | 2 | 0 | 2 | 100% concordant (malignant) |
| Total | 3 | 6 | 1 | 1 | 3 | 2 | 2 | 18 | 10 | 8 | Overall: 88.9% concordance (16/18), 11.1% discordant (2/18) |

**Concordance was defined as agreement between FNAC (benign vs malignant categories) and postoperative histopathology. Bethesda III yielded one discordant malignant outcome (false negative). Bethesda IV cases demonstrated a 45.5% malignancy rate, consistent with the BSRTC expected risk range. Abbreviations: FNAC: Fine-needle aspiration cytology; BSRTC: Bethesda System for Reporting Thyroid Cytopathology; AUS/FLUS: Atypia of undetermined significance / Follicular lesion of undetermined significance; FN: Follicular neoplasm; PTC: Papillary thyroid carcinoma; PTC micro: Papillary microcarcinoma. FVPTC: Follicular variant of papillary thyroid carcinoma**
